# Diagnostic Yield of Internal Medicine Referrals to Rheumatology: A Retrospective Single-Center Study

**DOI:** 10.3390/medicina62101943

**Published:** 2026-10-08

**Authors:** Pınar Akyüz Dağlı, Berkan Armağan, Hatice Ecem Konak, Sevgi Bilen Ayhan, Zeynep Özge Özdemir, Esra Maide Özel, Müge Büyükaksoy, Emine Sena Sözen, Ebru Atalar, Rezan Koçak Ulucaköy, Esra Kayacan Erdoğan, Serdar Can Güven, İsmail Doğan, Kevser Orhan, Yüksel Maraş, Orhan Küçükşahin, Ahmet Omma, Şükran Erten, Hakan Babaoğlu

**Affiliations:** 1 Department of Rheumatology, Ankara Bilkent City Hospital, 06800 Ankara, Turkeyhakanbabaoglu@gmail.com (H.B.); 2 Clinic of Internal Medicine, Ankara Bilkent City Hospital, 06800 Ankara, Turkey; 3 Department of Internal Medicine, Division of Rheumatology, Ankara City Hospital, Ankara Yıldırım Beyazıt University, 06800 Ankara, Turkey; 4 Department of Internal Medicine, Division of Rheumatology, Ankara City Hospital, University of Health Sciences, 06800 Ankara, Turkey

**Keywords:** rheumatology, referral and consultation, diagnostic yield, health care utilization, internal medicine

## Abstract

*Background and Objectives*: In Turkey, access to adult rheumatology outpatient clinics requires referral from another specialty, mostly internal medicine, and no standardized referral algorithms are in use. We estimated the diagnostic yield of new inflammatory rheumatic disease (IRD) diagnoses among internal medicine referrals who completed rheumatology evaluation and identified referral-stage characteristics associated with a new IRD diagnosis. *Materials and Methods*: In this retrospective cohort study, 2233 patients referred from internal medicine clinics to rheumatology outpatient clinics between 1 September and 30 November 2022 were screened. Patients with a prior diagnosis of IRD (*n* = 539) and those who did not attend their scheduled appointment (*n* = 732) were excluded, leaving 962 patients who completed rheumatology evaluation for analysis. Findings documented at referral were compared between patients with and without a new IRD diagnosis, and associations were examined with a clinically selected multivariable logistic regression model without laboratory variables, because laboratory tests had been ordered selectively. *Results*: Among the 962 patients who completed rheumatology evaluation, 141 (14.6%) received a new IRD diagnosis, corresponding to 6.3% of all 2233 referrals; the diagnostic status of the 732 patients who did not attend is unknown. In the multivariable model, swollen and tender joints were associated with a new IRD diagnosis (odds ratio [OR] 5.24, 95% confidence interval [CI] 3.17–8.68), whereas sicca symptoms (OR 1.45, 95% CI 0.83–2.55) and constitutional symptoms (OR 1.66, 95% CI 0.76–3.64) were not independently associated. *Conclusions*: Among patients referred from internal medicine to a tertiary rheumatology clinic who completed evaluation, approximately one in seven received a new IRD diagnosis. Referral appropriateness was not assessed, and the high non-attendance rate limits generalization to all referrals. Swollen and tender joints were the only referral characteristic independently associated with a new diagnosis. Whether structured referral criteria, medical education, or triage strategies improve the yield of referrals is a hypothesis for prospective evaluation.

## 1. Introduction

Inflammatory rheumatic diseases (IRDs) are associated with high morbidity and mortality, yet their course can be significantly improved with early diagnosis and appropriate treatment [1]. Rheumatology has become a critical specialty in the diagnosis and management of these complex, chronic, and sometimes life-threatening conditions that affect the joints, connective tissue, and internal organs. The introduction of targeted biologic therapies, advanced imaging modalities, and expanded autoantibody panels has improved the diagnosis and treatment of rheumatic diseases. Outpatient visits to rheumatology clinics, particularly due to the heterogeneous clinical spectrum of IRDs, are placing an increasingly heavy burden on healthcare systems worldwide.

The increasing prevalence of rheumatic diseases [2], the rapidly rising demand for rheumatology outpatient services, and the concentration of specialists predominantly in tertiary care centers have created a marked imbalance between supply and demand in adult rheumatology services. This situation has highlighted the need for innovative and evidence-based approaches aimed at reducing low-priority referrals and improving patient access. However, a considerable proportion of the patient load in rheumatology clinics consists of individuals without inflammatory rheumatic diseases. In previous reports, approximately one-third of referrals to rheumatologists were judged unnecessary or inappropriate [3,4]. Only 30–40% of patients referred to rheumatology receive a confirmed rheumatologic diagnosis, while the remaining cases are most often referred for musculoskeletal pain, degenerative joint disease, or nonspecific laboratory abnormalities [5,6]. The demand for rheumatology outpatient services has increased, creating challenges in aligning patient needs with the most appropriate level of care, which has contributed to prolonged waiting times and increased financial burden on the healthcare system. Non-priority or misdirected referrals consume limited specialist capacity and may prevent timely care for patients requiring urgent evaluation. Such referrals contribute to diagnostic delays, increase resource utilization, and result in a significant rise in healthcare expenditures [7]. Appropriate referrals to rheumatology can improve patient outcomes while also reducing costs through the optimal use of physician time, hospital resources, and laboratory services [6]. Despite increasing awareness in the literature and in practice regarding referral-related issues, relatively few studies have focused on inter-specialty referrals in hospital outpatient clinics and how these processes can be improved [7,8,9]. Most of these data indicate that the quality and timing of referral processes to rheumatology are at least as important as diagnosis and treatment in patient management.

In gatekeeper systems such as those in Turkey, access to subspecialties like rheumatology requires prior referral from an intermediary level. In the Turkish healthcare system, family physicians cannot directly refer patients to rheumatology outpatient clinics. Referrals to adult rheumatology services in tertiary care centers are predominantly made by internal medicine physicians through the National Centralized Appointment System. While this structure theoretically offers advantages in ensuring appropriate referrals and efficient use of resources, the absence of standardized algorithms and auditing mechanisms may lead to an increase in low-yield referrals. This challenge is not unique to Turkey but represents a universal difficulty observed in many healthcare systems adopting the gatekeeper model.

Internists serve as the first point of contact for individuals with suspected rheumatic disease, performing a detailed history, physical examination, and basic laboratory evaluation to determine the need for rheumatology referral. The lack of referral algorithms and monitoring mechanisms contributes to the growing patient load in subspecialty clinics. Large-scale and up-to-date data on the content, prioritization, and diagnostic outcomes of referrals to adult rheumatology also remain limited in the current literature.

In this retrospective study of referrals from internal medicine to a tertiary adult rheumatology center, the primary objective was to estimate the diagnostic yield of new IRD diagnoses among patients who completed rheumatology evaluation, and the secondary objective was to identify referral-stage characteristics associated with a new IRD diagnosis; potential applications for referral pathways are discussed.

## 2. Materials and Methods

### 2.1. Study Design and Population

**Figure 1:**
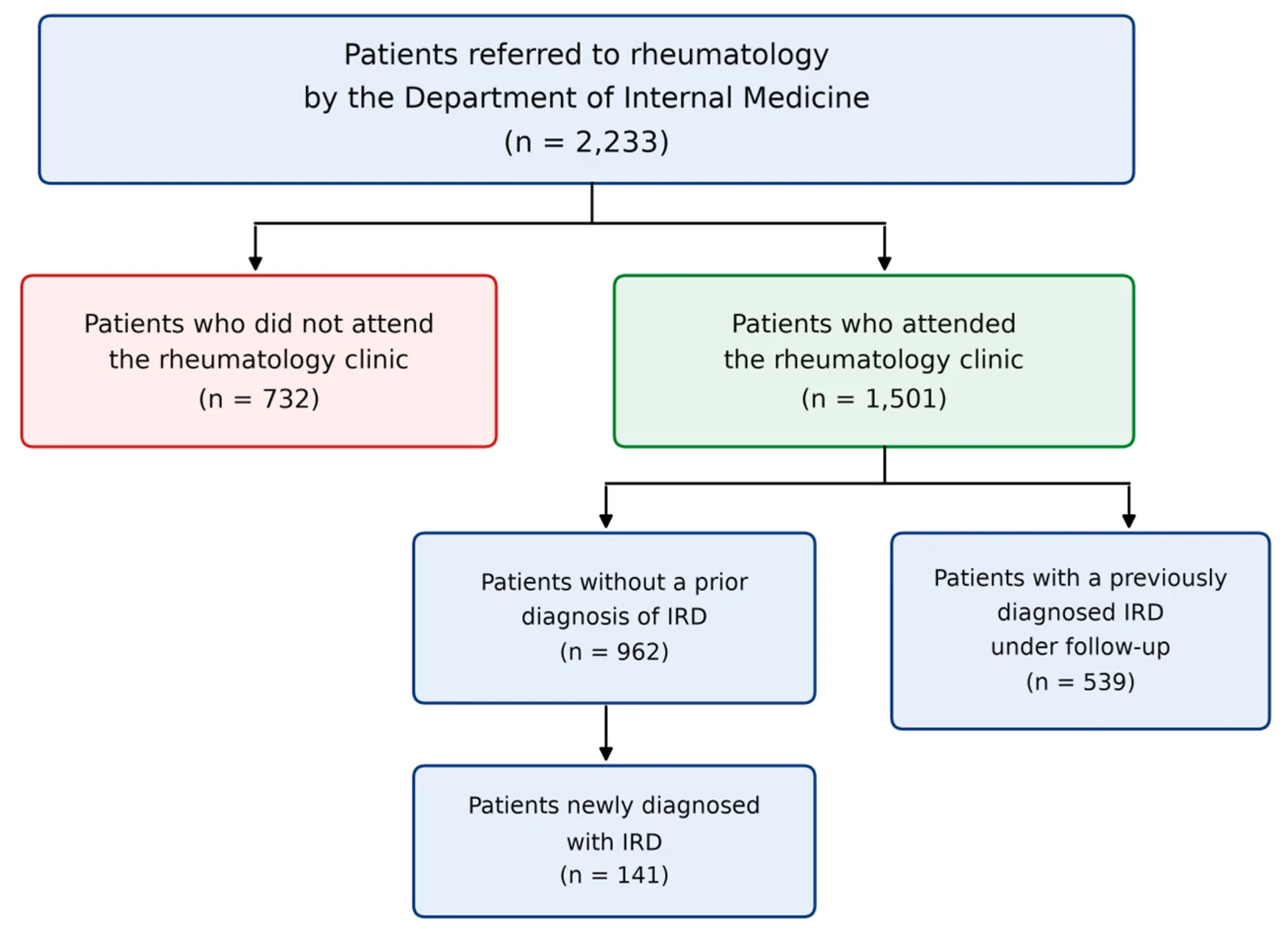
Flowchart of patients included in the study. IRD: Inflammatory rheumatic disease.

This retrospective cohort study included adult patients aged ≥18 years who were referred from the Department of Internal Medicine to the rheumatology outpatient clinics of a tertiary care hospital between 1 September and 30 November 2022. A total of 2233 referrals were screened. Patients who did not attend their rheumatology appointment or who had a prior diagnosis of IRD were excluded (Figure 1). The final study cohort consisted of 962 patients who completed a rheumatology evaluation. Because the diagnostic status of non-attenders is unknown, the diagnostic yield reported here refers to patients who completed evaluation rather than to all referrals.

IRD was defined as an inflammatory rheumatic disease diagnosed clinically by the evaluating rheumatologist on the basis of history, physical examination, laboratory tests, and imaging. IRD is used here as a broad umbrella term, because the new diagnoses were heterogeneous and included gout, axial spondyloarthritis, undifferentiated connective tissue disease, and seronegative arthritis. Classification criteria were not formally applied, and diagnoses were not independently adjudicated; the clinical diagnosis of a single rheumatologist was accepted as final. Final diagnoses were those recorded in the electronic medical record up to the date of data extraction in May 2023, six months after the end of the referral period, so that every patient had at least six months of potential follow-up after referral; no fixed follow-up window was applied. Undifferentiated connective tissue disease denoted clinical and serological features of a connective tissue disease that did not fit a defined entity, and seronegative arthritis denoted inflammatory arthritis without rheumatoid factor or anti-cyclic citrullinated peptide (anti-CCP) antibodies that was not classified as a specific disease. Because these two diagnoses are less firmly defined, the diagnostic yield was also calculated after their exclusion.

During the study period, internal medicine physicians did not use standardized referral forms or predefined referral algorithms when referring patients to the rheumatology outpatient clinics. Referrals were made based on routine clinical judgment and were recorded in the hospital’s electronic medical record system. Referral appropriateness was not formally assessed in this study. A referral that did not result in a new IRD diagnosis was classified as a low-yield referral rather than an inappropriate one, because specialist evaluation may have been warranted to exclude inflammatory disease.

All patients included in the study were covered by the national health insurance system, which provides access to outpatient and specialty care in Turkey.

### 2.2. Data Collection

For each patient, demographic data, clinical characteristics, laboratory findings, and imaging results were retrieved from electronic medical records. For patients without a prior diagnosis of IRD, the clinical, laboratory, and radiological reasons for referral, as well as any newly established rheumatologic diagnoses, were recorded. Diagnostic investigations performed during the initial internal medicine evaluation included a detailed medical history, physical examination, and basic laboratory tests, whereas further diagnostic assessments and confirmatory investigations were conducted during the rheumatology outpatient visit. Although some patients received a diagnosis at the initial rheumatology visit, others underwent additional investigations and short-term follow-up within the rheumatology clinic before a final diagnosis was established. Patients were subsequently classified into two groups: those with a new IRD diagnosis and those without (non-IRD group). All variables analyzed in this study (referral reasons, symptoms, examination findings, laboratory results, and symptom duration) were those documented by the referring internal medicine physician at the time of referral; investigations performed during the rheumatology evaluation were used only to establish the diagnosis and were not entered into the analyses. A review of rheumatologic symptoms was recorded as documented when the internal medicine note contained a systematic inquiry into rheumatologic symptoms beyond the presenting complaint, such as morning stiffness, joint swelling, sicca symptoms, Raynaud’s phenomenon, photosensitivity, oral or genital aphthae, and constitutional symptoms. Constitutional symptoms comprised fever, unintentional weight loss, fatigue, and night sweats. Symptom duration was abstracted from the referral documentation. Laboratory tests were ordered at the discretion of the referring physician, and the number of patients tested could not be retrieved from the records; laboratory findings are therefore reported as documented positive results among all patients in each group and do not represent test positivity rates.

### 2.3. Statistical Analysis

Comparisons between groups were performed using the chi-square test, Fisher’s exact test, or Student’s *t*-test, as appropriate. Factors associated with a new IRD diagnosis were examined by multivariable logistic regression in all 962 patients (141 outcome events). Because laboratory tests had been ordered selectively and the number of patients tested was unknown, laboratory variables were not included in the main model. The main model was a post hoc, clinically selected, laboratory-free model fitted without automated variable selection, with all variables entered simultaneously: age, sex, arthralgia, swollen and tender joints, chronic low back pain, sicca symptoms (xerostomia or xerophthalmia), constitutional symptoms, mucocutaneous lesions, and documented review of rheumatologic symptoms. These variables were chosen as referral features that commonly raise suspicion of an IRD and were recorded for every patient. Raynaud’s phenomenon and thrombosis were not included because each was present in only three patients with a new IRD diagnosis, and referral for an abnormal laboratory finding was not included because it depends on the same selective testing; the model had 15.7 events per variable. Multicollinearity (variance inflation factors), linearity of age in the logit (restricted cubic spline with four knots), separation, and influential observations (Cook’s distance, with re-estimation after excluding the ten most influential observations) were checked, and the estimates were compared with Firth penalized estimates. Calibration (Hosmer–Lemeshow test) and discrimination (area under the receiver operating characteristic curve [AUC], with a bootstrap 95% confidence interval from 2000 resamples) are reported for completeness; the model was intended to describe associations with a new IRD diagnosis, not to predict it. An exploratory model including laboratory variables is reported in Supplementary Table S1; it does not correct for selective testing or exposure misclassification, and its laboratory estimates are not interpreted as diagnostic associations. Its candidate variables were age, sex, documented review of rheumatologic symptoms, assessment of pain characteristics, referral for a clinical, laboratory, or imaging finding, swollen and tender joints, hand pain, foot pain, xerostomia, xerophthalmia, constitutional symptoms, thrombosis, elevated erythrocyte sedimentation rate (ESR), elevated C-reactive protein (CRP), elevated uric acid, anti-CCP positivity, extractable nuclear antigen (ENA) positivity, widespread pain, and rheumatoid factor positivity. The final model was obtained by backward stepwise selection (conditional method, as implemented in IBM SPSS Statistics, Version 27.0 (IBM Corp., Armonk, NY, USA); removal at *p* > 0.10). Because information on whether pain characteristics had been assessed was missing for 278 patients (28.9%), the model was fitted on the 684 patients with complete data (113 outcome events), giving 14 events per variable in the final model and 5.4 per candidate variable at entry. This model was also re-estimated with Firth penalized logistic regression in all 962 patients, with missing information on the assessment of pain characteristics coded as not assessed. Characteristics recorded at referral were compared between patients who did not attend (*n* = 732) and all patients who attended (*n* = 1501) with the same tests (Supplementary Table S2). Laboratory findings were not compared statistically, either between the IRD and non-IRD groups (Supplementary Table S3) or between non-attenders and attenders. Group comparisons and the exploratory stepwise model were performed with IBM SPSS Statistics 27 (IBM Corp., Armonk, NY, USA); the main model, its diagnostics, the Firth and full-cohort re-estimations, and the comparison of non-attenders were computed in Python 3.12 with statsmodels 0.15. A *p*-value < 0.05 was considered statistically significant.

## 3. Results

Of the 2233 patients referred from internal medicine to the rheumatology outpatient clinics, 70.9% were female. Of these, 732 (32.7%) did not attend their rheumatology appointment within the three-month follow-up period. Of the 1501 patients who attended, 539 (24.1% of all referrals) were already under rheumatology follow-up for an established IRD and were excluded, leaving 962 patients (43.1% of all referrals) who completed evaluation and were analyzed (Figure 1).

Patients who did not attend their rheumatology appointment did not differ significantly from all patients who attended (*n* = 1501) in terms of age or sex. Their characteristics recorded at referral are compared with those of all attenders in Supplementary Table S2: non-attenders less often had arthralgia, swollen and tender joints, low back pain, xerostomia, and xerophthalmia and were less often referred for an imaging finding, whereas the frequency of constitutional symptoms did not differ. Laboratory findings were not compared because tested denominators were unavailable. Their diagnostic status after referral is unknown. All patients were covered by the national health insurance system.

Of the 962 patients who completed evaluation, 141 (14.6%) received a new diagnosis of IRD. These 141 diagnoses represent 6.3% of all 2233 referrals; this is a descriptive proportion rather than a diagnostic yield for all referrals, because the diagnostic status of the 732 non-attenders is unknown. The demographic and clinical characteristics of patients and comparisons between the IRD and non-IRD groups are summarized in Table 1. There was no significant difference in mean age between groups, and female predominance was observed in both. Clinically, arthralgia was more frequent in the IRD group (76.6% vs. 67.7%, *p* = 0.035), with swollen and tender joints showing the most striking difference (29.8% vs. 6.9%, *p* < 0.001). Xerostomia (13.5% vs. 6.9%, *p* = 0.008) and constitutional symptoms (7.8% vs. 3.9%, *p* = 0.038) were also significantly more common in the IRD group. The time from symptom onset to rheumatology evaluation was similar between groups (*p* = 0.229).

In the laboratory-free multivariable model including all 962 patients (141 with a new IRD diagnosis), swollen and tender joints were associated with a new IRD diagnosis (OR: 5.24, 95% CI: 3.17–8.68, *p* < 0.001) (Table 2). Sicca symptoms (OR: 1.45, 95% CI: 0.83–2.55, *p* = 0.193), constitutional symptoms (OR: 1.66, 95% CI: 0.76–3.64, *p* = 0.201), documented review of rheumatologic symptoms (OR: 1.25, 95% CI: 0.82–1.91, *p* = 0.308), and the remaining variables were not independently associated with a new diagnosis. Variance inflation factors were at most 1.26, age showed no significant departure from linearity in the logit (*p* = 0.100), and there was no evidence of separation; excluding the ten most influential observations did not change the main association (swollen and tender joints: OR: 5.90, 95% CI: 3.53–9.84), and Firth penalized estimates were nearly identical (OR: 5.12, 95% CI: 3.10–8.46). The Hosmer–Lemeshow test did not indicate lack of fit (χ^2^ = 10.32, df = 8, *p* = 0.243), and the apparent AUC was 0.67 (95% CI 0.61–0.72).

**Figure 2:**
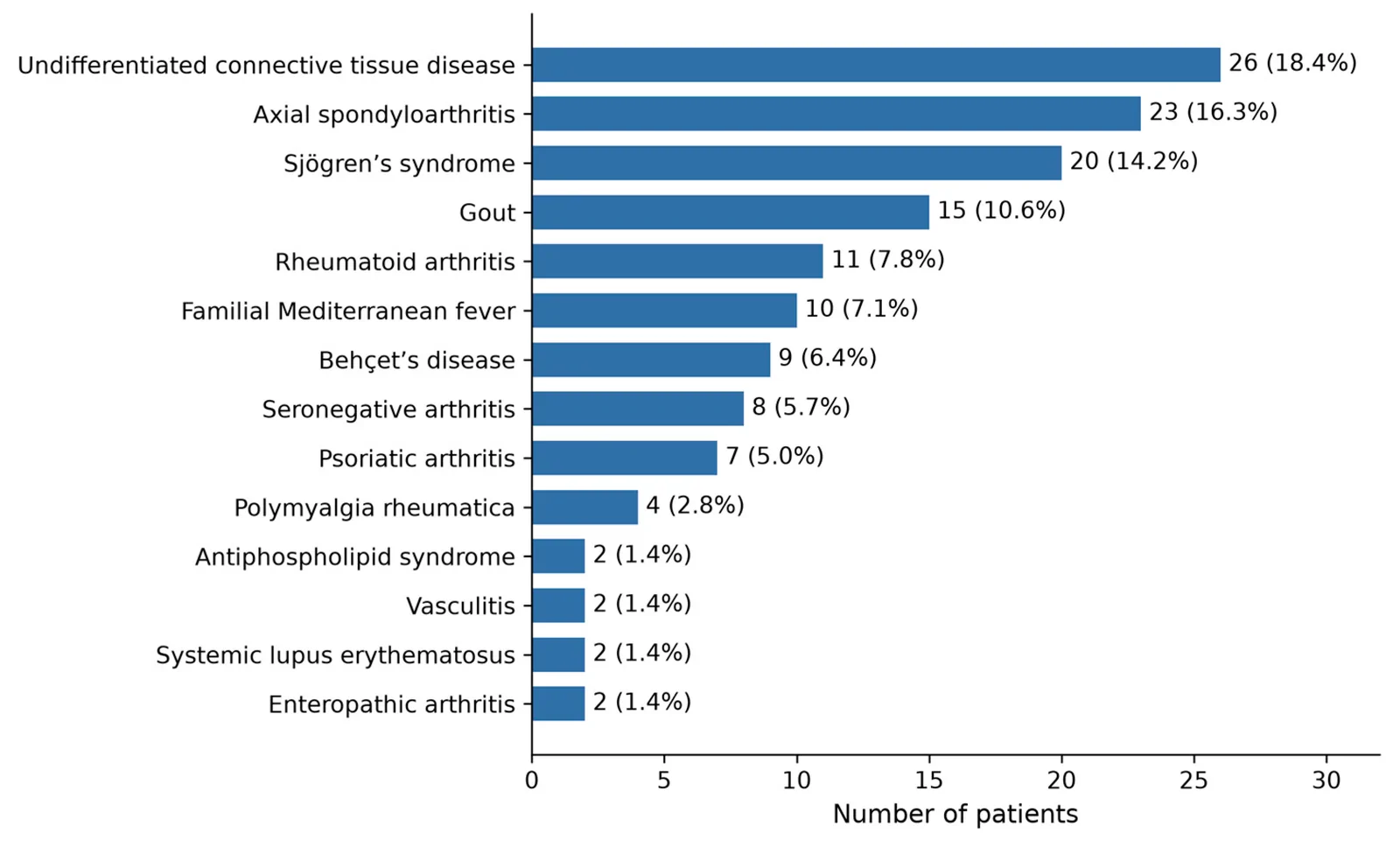
Distribution of the 141 patients with newly diagnosed IRDs according to disease type.

The distribution of newly diagnosed IRDs is illustrated in Figure 2. The most frequent diagnoses were undifferentiated connective tissue disease (*n* = 26), axial spondyloarthritis (*n* = 23), Sjögren’s syndrome (*n* = 20), gout (*n* = 15), rheumatoid arthritis (*n* = 11), familial Mediterranean fever (*n* = 10), Behçet’s disease (*n* = 9), seronegative arthritis (*n* = 8), psoriatic arthritis (*n* = 7), and polymyalgia rheumatica (*n* = 4). Less common conditions included enteropathic arthritis (*n* = 2), systemic lupus erythematosus (*n* = 2), vasculitis (*n* = 2), and antiphospholipid syndrome (*n* = 2). When undifferentiated connective tissue disease (*n* = 26) and seronegative arthritis (*n* = 8) were excluded, 107 patients (11.1%) had a new diagnosis of a more firmly defined IRD.

Laboratory tests were ordered selectively, and the number of patients tested is unknown; laboratory findings are therefore reported only as documented positive results among all patients in each group (Supplementary Table S3). Documented elevated CRP (42.6% vs. 17.8%), elevated ESR (20.6% vs. 9.5%), and anti-CCP positivity (7.8% vs. 1.6%) were more frequent in the IRD group, whereas documented antinuclear antibody (ANA) positivity was similar (28.4% vs. 26.3%). In the exploratory stepwise model that included laboratory variables (Supplementary Table S1), documented elevated CRP, elevated uric acid, and anti-CCP positivity were retained; because these estimates do not account for selective testing, they are not interpreted as diagnostic associations.

## 4. Discussion

In this study, 14.6% of patients referred from internal medicine to a tertiary rheumatology clinic in Turkey who completed evaluation received a new IRD diagnosis. This proportion is lower than the 30–60% reported in most published series, although differences in disease definitions, referral populations, and healthcare systems limit direct comparison. Because referral appropriateness was not assessed, a low yield does not by itself indicate that referrals were inappropriate; many patients may have been appropriately referred to exclude inflammatory disease. Nevertheless, most referrals did not result in a new IRD diagnosis, and such referrals add to the caseload of rheumatology clinics even when they are made to exclude inflammatory disease. Whether structured referral pathways would improve the use of specialized rheumatology services is a hypothesis that our data cannot test. Below, we separate observations in this cohort from evidence obtained in other healthcare systems.

Table 3 summarizes published estimates of diagnostic yield from other settings. These studies differ from ours in the outcome definition (differently defined inflammatory rheumatic disease categories, systemic autoimmune rheumatic disease, or inflammatory arthritis), in the referring physicians (general practitioners in most series versus internal medicine specialists in ours), and in the healthcare system; the denominators also differ, because not all studies excluded non-attenders or patients with an established diagnosis. The table is therefore intended as a contextual overview rather than a direct comparison, and the yield percentages should not be read as a ranking of referral quality across countries. With this caveat, reported yields from Canada [6] and Germany [10] were higher than ours, whereas the estimates from the Netherlands [11] and the United States [12] illustrate the wide variation across healthcare systems.

In the study by Hoffmann et al., more than half of patients referred to the rheumatology outpatient clinic received a rheumatologic diagnosis. The most frequent diagnoses were inflammatory joint diseases, connective tissue diseases, and vasculitides [13]. Another study from Turkey demonstrated that 35.7% of patients referred to rheumatology were diagnosed with inflammatory rheumatic diseases, with joint symptoms being the most common reason for referral [5].

A similar pattern has been described in pediatric rheumatology. In a large pediatric rheumatology center, most new referrals did not result in a rheumatologic diagnosis [14]. Poor concordance between the referral diagnosis and the definitive diagnosis established by rheumatologists has also been reported [13], and such discordance may contribute to diagnostic delays. Whether initial assessment of non-inflammatory musculoskeletal pain within other clinical services would preserve timely access for patients with suspected inflammatory disease has not been tested.

Clinical red flags commonly proposed for prioritized referral include persistent joint swelling, inflammatory back pain, prolonged morning stiffness, unexplained elevation of inflammatory markers, constitutional symptoms after exclusion of infection and malignancy, Raynaud’s phenomenon, sicca symptoms, recurrent oral or genital ulcers, photosensitive rashes in combination with musculoskeletal or systemic features, uveitis, and thrombotic events in young patients.

Evidence from other healthcare systems suggests that limited curricular allocation and clinical exposure to rheumatology during internal medicine residency may contribute to low-yield referrals [15,16]; training was not examined in this study. Internal medicine and family medicine residents have reported gaps in rheumatology training [17] and lower confidence in rheumatology than in other areas [18,19]. Whether more rheumatology training during residency would improve referral yield is a hypothesis for prospective evaluation.

In the laboratory-free multivariable model, swollen and tender joints documented at referral were the only characteristics independently associated with a new IRD diagnosis. Sicca symptoms, constitutional symptoms, and a documented review of rheumatologic symptoms were more frequent in the IRD group but were not independently associated after adjustment. Similarly, a cross-sectional study of 697 patients with arthralgia reported that the most important predictors of inflammatory arthritis were joint swelling/tenderness and morning stiffness lasting ≥1 h, whereas longer duration of arthralgia and fatigue were identified as negative predictors [12]. In that study, these findings were rarely documented in referral notes. Together, these observations support the hypothesis that systematic joint examination and its documentation before referral would help identify patients likely to have an IRD, which requires prospective evaluation. Workforce expansion alone may not improve yield: in one health system, the employment of an additional rheumatologist was followed by an increase in referrals judged inappropriate [4], although this observation may not apply to other settings.

In a scoping review, the best-performing triage tools for suspected inflammatory arthritis were those based solely on patient-reported questions [20]. In Canada, the Central Referral and Triage in Rheumatology program reduced waiting times for urgent and semi-urgent referrals and improved referral quality within two years [21], and applying the 2010 ACR/EULAR criteria in referral triage identified rheumatoid arthritis with a high positive predictive value and shortened waiting times [22]. None of these approaches have been evaluated in our setting, and their effect on referral yield in a system such as ours remains to be tested.

Median times from symptom onset to rheumatology consultation of 24 months [21] and 27.2 weeks [23] have been reported, with delays most pronounced at the primary care level. In our cohort, the time from symptom onset to rheumatology evaluation showed a wider distribution; approximately one-quarter of patients were assessed within the first two weeks, whereas approximately one-third experienced a delay of over 12 weeks. In a simulation of a maximum waiting time guarantee, a modest reduction in referral volume improved average waiting times within one year [24]; waiting times after referral were not measured in our study. Approximately one-third of our referred patients did not attend. Non-attendance may be related to symptom resolution, prolonged waiting times, or patient-related factors, and it could be more or less frequent among patients with IRD; the direction of any resulting bias therefore cannot be determined from our data.

Laboratory findings could not be analyzed as diagnostic associations in this study. Documented elevated CRP and anti-CCP positivity were more frequent in the IRD group, whereas documented ANA positivity was not (Supplementary Table S3), but these proportions are subject to verification bias and exposure misclassification, and the laboratory estimates of the exploratory model (Supplementary Table S1) share this limitation. Referral prompted by an isolated laboratory abnormality without supporting clinical features is nevertheless common. In a study of serological testing after a positive ANA result, errors were more frequent among non-rheumatology physicians than among rheumatologists [25]. In pediatric rheumatology, none of the 49 patients referred with positive laboratory results but no clinical symptoms had a rheumatologic disease [26]. Whether referral algorithms that promote targeted laboratory testing before referral improve diagnostic yield should be evaluated prospectively in studies that record which tests were performed.

This study has several limitations. First, its single-center and retrospective design limits the generalizability of the findings, and referral dynamics in a tertiary hospital may not reflect those of other regions or levels of care. Second, approximately one-third of referred patients did not attend their rheumatology appointment. Their characteristics at referral could be compared with those of all patients who attended (Supplementary Table S2), but their diagnostic status is unknown; the reported diagnostic yield therefore applies to patients who completed evaluation, and selection bias in either direction cannot be excluded. Third, referral appropriateness was not formally assessed, and detailed data on the completeness and content of referral notes were not available; a referral that did not result in a new IRD diagnosis cannot be assumed to have been inappropriate. Alternative diagnoses and management outcomes in the non-IRD group were not systematically captured and could not be analyzed. Fourth, diagnoses were clinical diagnoses made by the evaluating rheumatologist, follow-up after referral was at least six months but of variable length, and the IRD category is heterogeneous, ranging from gout to undifferentiated connective tissue disease; excluding the two least firmly defined diagnoses reduced the yield from 14.6% to 11.1%. Fifth, laboratory tests, including autoantibodies and uric acid, were ordered selectively by the referring physician, and the number of patients tested could not be retrieved; negative results and tests not performed could not be distinguished, so documented laboratory findings are subject to verification bias and exposure misclassification. Laboratory variables were therefore excluded from the main model, and the exploratory estimates in Supplementary Table S1 cannot be interpreted as diagnostic associations. Sixth, the variables of the main model were chosen post hoc on clinical grounds, and other reasonable selections were possible; the model describes associations, has modest discrimination, and was not developed or validated as a prediction tool. Seventh, variability in the quality of laboratory data and clinical notes may have influenced diagnostic accuracy. Eighth, healthcare costs, waiting times, and the effects of referral interventions were not measured, so the proposals discussed above are hypotheses for prospective evaluation. Finally, individual-level factors such as educational status, socioeconomic conditions, and patient preferences, which could affect access to care, were not analyzed.

## 5. Conclusions

In this single-center study, most referrals from internal medicine to rheumatology did not result in a new IRD diagnosis. In a laboratory-free model, swollen and tender joints documented at referral were the only characteristic independently associated with a new diagnosis; selectively ordered laboratory tests could not be evaluated as diagnostic markers. Prospective studies should evaluate whether structured referral criteria, triage strategies, rational use of laboratory testing, and educational interventions improve diagnostic yield, access, or resource use.

## Figures and Tables

**Table 1:** Demographic and clinical characteristics of patients referred to the rheumatology clinic.

Demographic and Clinical Features	Total *n* = 962	IRD *n* = 141	Non-IRD *n* = 821	*p* Value
Age (years), mean ± SD	47.6 ± 14.6	47.7 ± 16.3	47.5 ± 14.3	0.902
Female, *n* (%)	732 (76.1)	103 (73.0)	629 (76.6)	0.359
Smoking, *n* (%)	90 (9.4)	17 (12.1)	73 (8.9)	0.233
Any comorbidity, *n* (%)	480 (49.9)	74 (52.5)	406 (49.5)	0.506
Review of rheumatologic symptoms, *n* (%)	280 (29.1)	57 (40.4)	223 (27.2)	0.001
**Patients whose pain characteristics were evaluated,** ***n*** **(%)**	190 (19.8)	45 (31.9)	145 (17.7)	<0.001
Inflammatory pain Non-inflammatory pain Pain characteristics unclear	120 (63.2) 50 (26.3) 20 (10.5)	39 (86.7) 2 (4.4) 4 (8.9)	81 (55.9) 48 (33.1) 16 (11.0)	<0.001
Not recorded	278 (28.9)	28 (19.9)	250 (30.5)	
**Reasons for rheumatology consultation,** ***n*** **(%)**				
Clinical finding	561 (58.3)	96 (68.1)	465 (56.6)	0.011
Abnormal laboratory finding	437 (45.4)	84 (59.6)	353 (43.0)	<0.001
Abnormal imaging finding	56 (5.8)	15 (10.6)	41 (5.0)	0.008
**Chief symptoms of patients,** ***n*** **(%)**				
Arthralgia	664 (69.0)	108 (76.6)	556 (67.7)	0.035
Swollen and tender joints	99 (10.3)	42 (29.8)	57 (6.9)	<0.001
Widespread pain	146 (15.2)	17 (12.1)	129 (15.7)	0.264
Hand pain	162 (16.8)	31 (22.0)	131 (16.0)	0.077
Foot pain	122 (12.7)	27 (19.1)	95 (11.6)	0.012
Knee pain	112 (11.6)	14 (9.9)	98 (11.9)	0.492
Leg pain	63 (6.5)	10 (7.1)	53 (6.5)	0.778
Low back pain	163 (16.9)	22 (15.6)	141 (17.2)	0.646
Hip pain	44 (4.6)	4 (2.8)	40 (4.9)	0.285
Shoulder pain	24 (2.5)	5 (3.5)	19 (2.3)	0.386
Headache	7 (0.7)	1 (0.7)	6 (0.7)	>0.999
Xerostomia	76 (7.9)	19 (13.5)	57 (6.9)	0.008
Xerophthalmia	67 (7.0)	15 (10.6)	52 (6.3)	0.064
Conjunctival hyperemia	14 (1.5)	3 (2.1)	11 (1.3)	0.505
Constitutional symptoms	43 (4.5)	11 (7.8)	32 (3.9)	0.038
**Fatigue**	104 (10.8)	17 (12.1)	87 (10.6)	0.606
Lymphadenopathy	1 (0.1)	0 (0)	1 (0.1)	>0.999
Panniculitis	2 (0.2)	0 (0)	2 (0.2)	>0.999
Raynaud’s phenomenon	24 (2.5)	3 (2.1)	21 (2.6)	>0.999
Thrombosis	9 (0.9)	3 (2.1)	6 (0.7)	0.133
Recurrent pregnancy loss	13 (1.4)	1 (0.7)	12 (1.5)	0.705
Recurrent abdominal pain	26 (2.7)	5 (3.5)	21 (2.6)	0.570
Recurrent fever	7 (0.7)	1 (0.7)	6 (0.7)	>0.999
Recurrent pericarditis	3 (0.3)	0 (0)	3 (0.4)	>0.999
Mucocutaneous lesions	72 (7.5)	12 (8.5)	60 (7.3)	0.601
**The duration of time from symptom onset to rheumatology examination**				
<2 weeks 2–12 weeks >12 weeks	236 (24.5) 407 (42.3) 319 (33.2)	39 (27.7) 64 (45.3) 38 (27.0)	197 (24.0) 343 (41.8) 281 (34.2)	0.229

Reasons for rheumatology consultation are not mutually exclusive. Documented laboratory findings at referral are shown in Supplementary Table S3.

**Table 2:** Factors associated with a new IRD diagnosis (laboratory-free multivariable logistic regression, *n* = 962).

Variable	Odds Ratio (95% CI) *	*p* Value
Age (per year)	1.00 (0.98–1.01)	0.685
Female sex	0.88 (0.57–1.34)	0.542
Arthralgia	1.03 (0.63–1.67)	0.906
Swollen and tender joint (s)	5.24 (3.17–8.68)	<0.001
Chronic low back pain	1.04 (0.60–1.78)	0.899
Sicca symptoms	1.45 (0.83–2.55)	0.193
Constitutional symptoms	1.66 (0.76–3.64)	0.201
Mucocutaneous lesions	1.21 (0.60–2.44)	0.585
Documented review of rheumatologic symptoms	1.25 (0.82–1.91)	0.308

* CI: confidence interval. Post hoc, clinically selected, laboratory-free model including all 962 patients (141 with a new IRD diagnosis); all nine variables were entered simultaneously without automated variable selection. Sicca symptoms: xerostomia or xerophthalmia. The stepwise model including laboratory variables is shown as an exploratory analysis in Supplementary Table S1.

**Table 3:** Reported diagnostic yield of rheumatology referrals in selected studies (contextual overview; not directly comparable).

Study/Country	Sample Size	Outcome Definition	Diagnostic Yield (%)	Referral Source/Healthcare System
Present study (Turkey, Ankara)	962 patients who completed evaluation (of 2233 referrals)	New IRD diagnosis (patients with established IRD excluded)	14.6	Internal medicine specialists (tertiary center)
Kutluk & Kaçar (Turkey, Antalya) [5]	1263	IRD	35.7	Internal medicine, orthopedics, physical medicine & rehabilitation, other specialties
Germany [10]	1782	IRD	40.3	General practitioners (gatekeeper system)
Canada [6]	2430	SARD	31.0	General practitioners and internal medicine
The Netherlands [11]	250	IRD	22.0	General practitioners (mandatory gatekeeper)
USA [12]	697	IA	25.7	Various specialties; early arthritis clinics

Abbreviations: IRD: inflammatory rheumatic disease; SARD: systemic autoimmune rheumatic disease; IA: inflammatory arthritis. Studies differ in outcome definition, referral source, denominator, and healthcare system; the percentages are not directly comparable.

## Data Availability

The data supporting the findings of this study are not publicly available due to ethical and privacy considerations related to human subjects but are available from the corresponding author upon reasonable request.

## Supplementary Materials

The following supporting information can be downloaded at https://www.mdpi.com/article/10.3390/medicina62101943/s1, Table S1: Exploratory multivariable logistic regression including laboratory variables for a new IRD diagnosis (backward stepwise model) and its sensitivity analyses; Table S2: Characteristics recorded at referral: patients who did not attend the rheumatology appointment versus all patients who attended; Table S3: Documented positive laboratory findings at referral: exploratory comparison; tested denominators unavailable.
