# Supplementary material for: Diagnostic Yield of Internal Medicine Referrals to Rheumatology: A Retrospective Single-Center Study
Source: Medicina (Kaunas). 2026 Oct 8;62(10):1943. doi: 10.3390/medicina62101943 (PMC13707323; doi:10.3390/medicina62101943)
Supplement: Supplementary file 1 [file medicina-62-01943-s001.zip › medicina-4545798-supplementary.pdf]

Supplementary Table S1. Exploratory multivariable logistic regression including laboratory variables for a new IRD diagnosis (backward stepwise model) and its sensitivity analyses

Variable	Model 1: stepwise model including laboratory variables (complete-case) (n = 684; events = 113)		Model 2: Firth penalized regression (complete-case) (n = 684; events = 113)		Model 3: all patients, missing pain-assessment information coded as not assessed (n = 962; events = 141)	
	OR (95% CI)	p	OR (95% CI)	p	OR (95% CI)	p
Age (per year)	0.98 (0.96–0.99)	0.009	0.98 (0.96–1.00)	0.010	0.99 (0.98–1.00)	0.126
Pain characteristics assessed	1.63 (1.00–2.65)	0.049	1.62 (1.00–2.62)	0.050	1.75 (1.12–2.75)	0.014
Swollen and tender joint(s)	4.38 (2.57–7.46)	<0.001	4.26 (2.51–7.23)	<0.001	4.30 (2.59–7.12)	<0.001
Xerostomia	2.32 (1.18–4.58)	0.015	2.31 (1.18–4.53)	0.015	1.92 (1.03–3.56)	0.039
Thrombosis	9.59 (1.24–74.37)	0.031	9.26 (1.20–71.67)	0.033	4.02 (0.93–17.35)	0.062
Increased C-reactive protein	4.17 (2.57–6.79)	<0.001	4.07 (2.51–6.60)	<0.001	3.54 (2.34–5.36)	<0.001
Increased uric acid levels	7.08 (1.93–26.02)	0.003	6.82 (1.87–24.87)	0.004	7.58 (2.34–24.56)	<0.001
Anti-CCP positivity	4.61 (1.64–12.94)	0.004	4.50 (1.61–12.58)	0.004	3.38 (1.29–8.89)	0.013

OR: odds ratio; CI: confidence interval; IRD: inflammatory rheumatic disease; CCP: cyclic citrullinated peptide. In Model 1, candidate variables were age, sex, and 19 clinical and laboratory variables; the final model was obtained by backward stepwise selection (removal at $p > 0.10$). Because information on whether pain characteristics had been assessed was missing for 278 of 962 patients, Models 1 and 2 are restricted to the 684 patients with complete data. Model 2 re-estimates the same model with Firth penalized likelihood (Wald confidence intervals). Model 3 includes all 962 patients, with missing pain-assessment information coded as not assessed. In the complete-case subset, thrombosis was present in four patients, elevated uric acid in 15, and anti-CCP positivity in 21. Model 1 reproduced its odds ratios to within 0.01. Variance inflation factors in Model 1 were all below 1.1.

Supplementary Table S2. Characteristics recorded at referral: patients who did not attend the rheumatology appointment versus all patients who attended

Characteristic	Did not attend (n = 732)	Attended (n = 1501)	p value
Age (years), mean ± SD	46.6 ± 14.6	47.5 ± 14.8	0.188
Female, n (%)	505 (69.0)	1078 (71.8)	0.183
Arthralgia, n (%)	371 (50.7)	875 (58.3)	<0.001
Swollen and tender joints, n (%)	55 (7.5)	156 (10.4)	0.035
Widespread pain, n (%)	72 (9.8)	175 (11.7)	0.224
Low back pain, n (%)	74 (10.1)	213 (14.2)	0.008
Xerostomia, n (%)	23 (3.1)	103 (6.9)	<0.001
Xerophthalmia, n (%)	27 (3.7)	94 (6.3)	0.015
Constitutional symptoms, n (%)	24 (3.3)	55 (3.7)	0.733
Raynaud's phenomenon, n (%)	16 (2.2)	35 (2.3)	0.947
Review of rheumatologic symptoms documented, n (%)	162 (22.1)	368 (24.5)	0.234
Referral for a clinical finding, n (%)	369 (50.4)	823 (54.8)	0.055
Referral for an abnormal laboratory finding, n (%)	235 (32.1)	543 (36.2)	0.065
Referral for an abnormal imaging finding, n (%)	21 (2.9)	80 (5.3)	0.012

SD: standard deviation. Data are those recorded in the referral and internal medicine records; the diagnostic status of patients who did not attend is unknown. Both groups may include patients with an established rheumatic diagnosis (539 among attenders were excluded from the analysis cohort for this reason); the comparison therefore describes the referred populations, not the analysis cohort. Age was compared with Student's t-test and categorical variables with the chi-square test or Fisher's exact test.

Supplementary Table S3. Documented positive laboratory findings at referral: exploratory comparison; tested denominators unavailable

Documented positive finding, n/N (%)	Total (N = 962)	IRD (N = 141)	Non-IRD (N = 821)
Increased erythrocyte sedimentation rate	107/962 (11.1)	29/141 (20.6)	78/821 (9.5)
Increased C-reactive protein	206/962 (21.4)	60/141 (42.6)	146/821 (17.8)
Increased uric acid levels	17/962 (1.8)	10/141 (7.1)	7/821 (0.9)
Rheumatoid factor positivity	108/962 (11.2)	21/141 (14.9)	87/821 (10.6)
Anti-CCP positivity	24/962 (2.5)	11/141 (7.8)	13/821 (1.6)
Antinuclear antibody positivity	256/962 (26.6)	40/141 (28.4)	216/821 (26.3)
Abnormal extractable nuclear antigen	107/962 (11.1)	23/141 (16.3)	84/821 (10.2)

IRD: inflammatory rheumatic disease; CCP: cyclic citrullinated peptide. Values are the numbers of patients with a documented positive result divided by all patients in each group. Laboratory tests were ordered at the discretion of the referring physician, a patient without a documented positive result may have had a negative test or no test. The percentages are therefore not test positivity rates, and no statistical tests were performed.
